# Nodulation of *Retama monosperma* by *Ensifer aridi* in an Abandonned Lead Mine Soils in Eastern Morocco

**DOI:** 10.3389/fmicb.2019.01456

**Published:** 2019-07-23

**Authors:** Hanane Lamin, Soufiane Alami, Omar Bouhnik, Salma ElFaik, Hanaa Abdelmoumen, Eulogio J. Bedmar, Mustapha Missbah-El Idrissi

**Affiliations:** ^1^Center for Biotechnology, Biodiversity and Environment, Faculty of Sciences, Mohammed V University, Rabat, Morocco; ^2^Department of Soil Microbiology and Symbiotic Systems, Estación Experimental del Zaidín, Consejo Superior de Investigaciones Científicas, Granada, Spain

**Keywords:** *Retama monosperma*, mining site tailings, *Ensifer aridi*, housekeeping genes, symbiotic genes

## Abstract

Millions tons of lead and zinc wastes from the abandoned Touissit mine are stored in the open air as dikes in the vicinity of the villages in Eastern Morocco and pose a real danger to both the environment and local populations. To prevent the movement of minerals to the nearby villages and limit the damages to the environment and health, we proposed the nitrogen-fixing leguminous shrub *Retama monosperma*, as a model plant to use for phytostabilization experimentations. This plant species is known by its ability to grow in hard climatic conditions and in heavy metals contaminated soils. The isolation of bacterial strains nodulating *R. monosperma* in the abandoned mine soils will permit the selection of rhizobia to inoculate young plant seedlings before their use for the phytostabilization of the mine tailings. In this work, 44 bacteria were isolated from the root nodules of *R. Monosperma* grown in the Touissit abandoned mine. Twenty-four isolates were considered as true rhizobia as they possess a copy of the nodC symbiotic gene and were able to renodulate their original host. The phenotypic characterization showed that all the strains are tolerant *in vitro* to different concentrations of heavy metals. The analysis of the 16S rRNA sequences of two selected representative strains showed they were related to different strains of *Ensifer aridi* isolated from different legumes in three continents deserts. The *glnII*, *recA*, and *gyrB* housekeeping genes analysis confirmed the affiliation of the strains to *E. aridi*. Moreover, the phylogenic analysis of *nodA*, *nodC*, and *nifH* symbiotic genes showed that the strains are more related to *E. aridi* JNVUTP6 species isolated from *Tephrosia purpurea* root nodules in the Thar Desert in India. To our knowledge, this is the first report about the isolation of *E. aridi* from *R. monosperma* root nodules.

## Introduction

The Touissit lead-zinc district is located in the south of Oujda (Eastern Morocco), straddling the Moroccan-Algerian border. The mining vocation of this region goes back to the beginning of this century, following prospections undertaken since 1906. However, such as other lead mines in Morocco, it was abandoned in 2002 since the price of this lead and zinc felt down at international level (Smouni et al., 2010). The wastes generated by mineral extraction were maintained in tailing ponds without any rehabilitation plan. These tailings are rich in heavy metals such as lead and zinc which contaminates the surrounding agricultural fields (Argane et al., 2014).

Metal trace elements contaminate surface water resources as well as agricultural products in surrounding areas. This scourge threatens the health of living beings and confirms hence the need for a quick solution. Several studies have shown the effectiveness of legumes utilization for the phytostabilization of degraded soils contaminated by different metallic trace elements (Foulkes, 2000; Gerhardt et al., 2009).

The efficiency of bioremediation depends mainly on the type of plant used and its ability to accumulate heavy metals pollutants, and on the bacterial partner and its ability to tolerate the toxicity of metal elements, lack of important nutrients and the abiotic stresses (El Aafi et al., 2012).

Several studies have shown the tolerance and resistance of rhizobia to different metal stresses (Jaishankar et al., 2014). These bacteria can colonize the soils of lead-rich mine sites. This metal is an environmental pollutant that has no known biological role (Muñoz Vallés et al., 2013). Lead is very dangerous to human health by inducing neurological and renal damages, it disturbs the blood pressure, and reproduction especially in humans and long-term exposure to this metallic element may even cause cancer (Naik et al., 2013; Najeeb et al., 2017). This metal also disrupts the biological processes of plants by replacing vital elements for the plant such as zinc, copper, or manganese (Najeeb et al., 2017), it can induce the total inhibition of plant growth by disrupting its vital processes such as the photosynthetic path and chlorophyllian chain, which are disturbed by damages of the membrane lipid layer and therefore the disruption of ion absorption.

Mining sites are known for degraded and poor soils lacking vital elements such as nitrogen and organic matter. The indigenous plants growing in the mining regions may be considered as naturally tolerant and may constitute an ecological solution for the phytostabilization of local mines soils.

*Retama monosperma* is a shrubby legume, native to North Africa and Southern Spain, with a large distribution in Morocco (Muñoz Vallés et al., 2013). It is known for its ability to fix atmospheric nitrogen in symbiosis with rhizobia, which strengthens its ability to tolerate extreme conditions, thus playing a key role in ecosystem restoration, desertification control and dune stabilization (Allen and Allen, 1981).

*Retama* species increases system’s forage productivity (Rivest et al., 2011) as they facilitate the growth of herbaceous plants growing in its understory, constituting “islands of fertility,” which are points of high biological activity (Pugnaire et al., 1996). Soil structure is hence stabilized by the roots, and by higher litter and organic matter content which create a microhabitat surrounding the *R. monosperma* canopy and participate to the retention of more moisture in the soil and leaching losses reduction (El-Bana et al., 2002; Flores and Jurado, 2003; Pugnaire et al., 2004; Dellafiore et al., 2008). The vegetation growing under *Retama* canopy increases its productivity as it benefits from the micro-environmental conditions and the nutrition *via* litter fall (López-Pintor et al., 2003; Muñoz Vallés et al., 2011). It can also be used for the phytostabilization of degraded soils (Caravaca et al., 2003) as well as soils contaminated by heavy metals and mining sites.

As a legume, *Retama* species have the ability to fix nitrogen by establishing a symbiosis with soil bacteria commonly called rhizobia. The main legume rhizobia belong to alpha and beta sub classes of the Proteobacteria. Rhizobial genera include *Agrobacterium*, *Allorhizobium*, *Aminobacter*, *Azorhizobium*, *Bradyrhizobium*, *Cupriavidus*, *Mesorhizobium*, *Methylobacterium*, *Microvirga*, *Ochrobactrum*, *Phyllobacterium*, *Rhizobium*, *Neorhizobium*, *Pararhizobium*, *Shinella*, *Ciceribacter*, and *Ensifer* (syn. *Sinorhizobium*) (Mousavi et al., 2014).

*Bradyrhizobium* is considered so far as the main genus of symbiotic nitrogen-fixing bacteria associated with *Retama* species (Rodríguez-Echeverría et al., 2003; Boulila et al., 2009; Guerrouj et al., 2013b). Previous studies of *Retama* rhizobia have shown that this plant establishes a symbiotic relationship exclusively with species of the genus *Bradyrhizobium*. Rodríguez-Echeverría et al. (2003) isolated some bacteria from nodules of *R. sphaerocarpa* in central Spain and affiliated them within the genus *Bradyrhizobium*. These isolates were classified later as *B. canariense* (Ruiz-Díez et al., 2008). The *R. sphaerocarpa* nodulating bacteria isolated by Boulila et al. (2009) constituted a new phylogenetic clade in the genus *Bradyrhizobium*. Guerrouj et al. (2013b) described *B. retamae*, the first species of *Bradyrhizobium* genus isolated from *R. monosperma*, in the subhumid area of Saidia in Northern Morocco, which was able to nodulate *R. sphaerocarpa* but not *Glycine max*.

However, Mahdhi et al. (2008) reported the nodulation of *Retama raetam* in arid zones in Tunisia by fast growing isolates that were close to *Sinorhizobium kostiense*, *S. meliloti*, and *Sinorhizobium* sp. and some isolates were related to *Rhizobium leguminosarum*, *Rhizobium sullae*, and *Rhizobium* sp.

The objective of the present study was the characterization of the fast growing symbiotic bacteria isolated from *R. monosperma* grown in soils of the abandoned mining site of Touissit in the arid Eastern area of Morocco. This characterization was based on a molecular analysis of symbiotic and housekeeping genes of the isolates, to determine their genetic diversity.

## Materials and Methods

### Isolation of Bacteria and Culture Conditions

Forty-four bacteria were isolated from the root nodules of *R. monosperma* plants collected from ten young plants grown in lead (Pb) tailings soil. The soils were sampled in the abandoned mining residues of Touissit, in the Eastern area of Morocco, at the frontiers border with Algeria (34°28′ 26 N – 001°46 19′ W) situated at 1148 m of altitude. This area is submitted to a double stress; the climate is arid with rare and random rainfall (90–100 mm/year) and the skeletal soils are neutral and contaminated with lead and zinc (1250–3300 mg/kg).

Nodules were washed under running tap water; then surface sterilized by immersion in 0.1% HgCl_2_ for 2 min, and finally washed thoroughly with sterile distilled water. Nodules were placed independently in Petri dishes and crushed in a drop of sterile water with a sterile glass.

The resulting suspension was streaked onto Petri dishes containing yeast extract-mannitol (YEM) medium (Vincent, 1970) supplemented with 0.0025% (w/v) Congo red. After incubation of the plates at 28°C for 10 days, colony forming units which represented all of the colony types that could be distinguished by microscopic observation of living cells were chosen. After identification, all rhizobial strains used in this study were routinely grown in Tryptone yeast-extract (TY) medium (Beringer, 1974).

### Heavy Metals Tolerance Assays

The intrinsic heavy metal resistance of the isolates was determined on TY agar medium. To this purpose, we prepared different media containing TY amended with each of the following heavy metals at the mentioned concentrations (in μg ml^–1^) HgCl_2_ (5); ZnCl_2_ (500; 1000); Pb-acetate (50, 300, 500 and 1000); MnCl_2_.4H_2_O (250 and 500); MgSO_4_ (500 and 1000); BaCl_2_, 2H_2_O (1000), CoSO4 (100); and FeCl_3_ (250). As the strains were isolated from mine tailings rich in zinc and lead, we tested different concentrations of these two metals on the growth of the strains. The strains were streaked on the different solid media and the Petri plates were incubated at 28°C for 7 days and any growth of the strains was considered a positive reaction.

### DNA Extraction, PCR Amplifications, and Sequencing

For DNA extraction and PCR amplifications, genomic DNA was isolated from bacterial cells as previously described (Guerrouj et al., 2013a). The quantity of DNA was determined by using a NanoDrop spectrophotometer (NanoDrop ND2000/2000c, Thermo Fisher Scientific, United States).

Rep-PCR (repetitive extragenic palindromic polymerase chain reaction) were performed using primers REP1 R-1 and REP2-I according to de Bruijn (1992), to reduce the number of strains and avoid any duplicates or clonality.

The DNA template was denatured for 1 min at 95°C, and PCR was carried out for 35 cycles (95°C for 15 s, 40°C for 15 s, and 72°C for 10 s), with a final elongation step at 72°C for 1 min, PCR was performed using a MyTaq Mix, following manufacturer’s specifications (Bioline Reagents Ltd.). PCR products were analyzed by horizontal electrophoresis in 2% agarose (Bioline) gels stained with ethidium bromide (EtBr) to a final concentration of 0.4% (p/w) (usually 2.5 μl of lab stock solution per 100 mL gel) in 1× Tris-acetate-EDTA (TAE) buffer at 70 V for 3 h. The profiles were photographed with ENDURO GDS Gel Documentation System. Comparative analysis of electrophoretic rep-PCR (enterobacterial repetitive intergenic consensus PCR) patterns was performed with GelCompar II software (version 2.5 Applied Maths, Belgium) by using UPGMA (Unweighted Pair Group Method with Arithmetic Averages).

PCR amplifications of 16S rRNA gene fragments were done using the two universal primers fD1 and rD1 previously described (Weisburg et al., 1991). Amplification products were checked by horizontal electrophoresis in 1% agarose (Bioline) gels stained with ethidium bromide (EtBr) to a final concentration of 0.4% (p/w) (usually 2.5 μl of lab stock solution per 100 mL gel) in TAE buffer at 70 V for 1 h, finally the gels were photographed under UV light.

The primer pairs recAf/recAr (Stepkowski et al., 2005), TSglnIIf/TSglnIIr and gyrB343F/gyrB1043R were used for amplification of *recA*, *gyrB*, and *glnII* genes, respectively, as described by Martens et al. (2007, 2008).

For the amplification of *nodA*, *nodC*, and *nifH* symbiotic genes, we used the primers nodA1F/nodAb1r; nodCFn/nodCi; nifHf/nifHi as described by Martens et al. (2007, 2008). The sequences obtained were compared with those from GenBank using the BLASTN program (Altschul et al., 1990). They were aligned using MEGA 7 software (Kumar et al., 2016). Distances calculated according to Kimura’s two-parameter model (Kimura, 1980) were used to infer phylogenetic trees with the neighbor-joining analysis (Saitou and Nei, 1987) with MEGA 7 software (Kumar et al., 2016). The sequences were compared to those of several *Ensifer* species validly published using MEGA 7 (Kumar et al., 2016). The strain *B. retamae* Ro9^T^ was used as an external reference taxon in the construction of the phylogenetic trees of the 16S rDNA and the housekeeping genes. Accession numbers of the nucleotide sequences of the *Ensifer* strains used in this study are shown in the figure trees.

For DNA sequencing, amplification products were purified using the PCR product purification system of Qiagen, and subjected to cycle sequencing using the same primers as for PCR amplification, with ABI Prism Dye Chemistry, and analyzed with a 3130 × l automatic sequencer at the sequencing facilities of Experimental Station of Zaidín, CSIC, in Granada, Spain for the 16S rDNA, and the other genes were sequenced at the sequencing facilities of National Centre for Scientific and Technical Research (CNRST) in Rabat (Morocco).

## Results

To test the use of *R. monosperma* for the stabilization of the lead mine tailings, the seedlings were grown in different soils of the Touissit abandoned mine area, in oriental Morocco. The lead (Pb) concentrations in these soils are higher than 3 g/kg of soil (Smouni et al., 2010). After 4 months of vigorous growth, the plants were recovered and checked for root nodulation and 44 bacteria were isolated.

**FIGURE 1:**
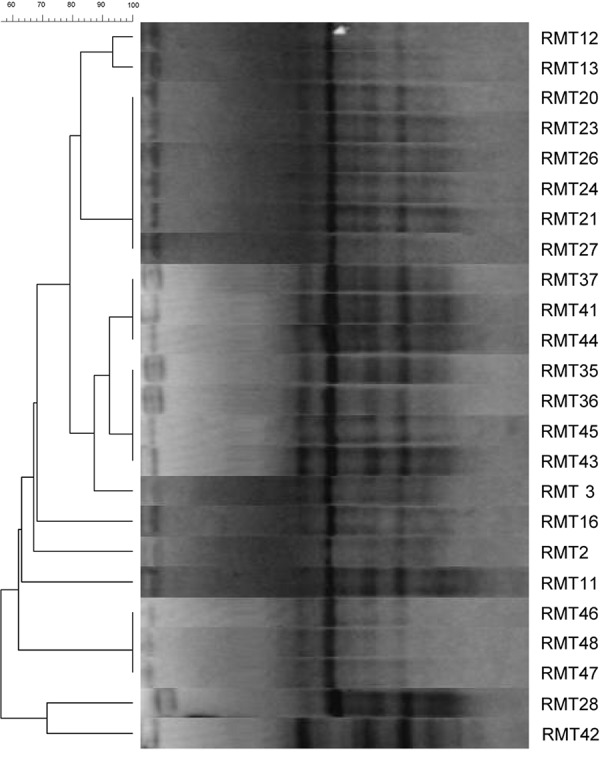
Dendrogram generated using Gelcompar II, by UPGMA clustering from REP-PCR fingerprinting of 24 bacterial strains isolated from the root nodules of *R. monosperma* grown in Touissit abandoned mine tailings.

The overall diversity of 44 isolates nodulating *R. monosperma* obtained in this study was firstly determined by REP–PCR DNA fingerprinting (Figure 1). This method allows also the prevention of duplication of the isolates. The 44 isolates were reduced to 29 different fingerprints, i.e., 29 strains (Table 1).

**TABLE 1:** Denomination of isolates and strains isolated from *R. monosperma* root nodules.

**Total bacteria Isolated^a^**	**Non-nodulating isolates^b^**	**Nodulating isolates^c^**	**Nodulating strains retained^d^**	**Identified strains^e^**
RMT1, RMT2, RMT3, RMT4, RMT5, RMT6, RMT7, RMT8, RMT9, RMT11, RMT12, RMT13, RMT14, RMT15, RMT16, RMT17, RMT18, RMT19, RMT20, RMT21, RMT23, RMT24, RMT26, RMT27, RMT28 RMT30, RMT31, RMT32, RMT33, RMT34, RMT35, RMT36, RMT37, RMT39, RMT40, RMT41, RMT42, RMT43, RMT44, RMT45, RMT46, RMT47, RMT48, RMT49	RMT1, RMT4, RMT5, RMT6, RMT7, RMT8, RMT9, RMT14, RMT15, RMT17, RMT18, RMT19, RMT30, RMT31, RMT32, RMT33, RMT34, RMT39, RMT40, RMT49	RMT12, RMT13, RMT20, RMT23, RMT26, RMT24, RMT21, RMT27, RMT37, RMT41, RMT44, RMT35, RMT36, RMT45, RMT43, RMT3, RMT16, RMT2, RMT11, RMT46, RMT48, RMT47, RMT28, RMT42	RMT2, RMT3, RMT11, RMT12, RMT16, RMT21, RMT36, RMT41, RMT42, RMT46	RMT3, RMT46

*^*a*^All the bacteria isolated from the root nodules of *R. monosperma*. ^*b*^All the strains that do not possess *nodC* gene copy and unable to renodulate *R. monosperma*. ^*c*^All the isolates that possess a *nodC* gene copy and were able to renodulate *R. monosperma*. ^*d*^All the strains defined by REP-PCR fingerprinting. ^*e*^The two representative strains selected following their differential level of tolerance to Pb-acetate.*

This method is highly discriminating and enables identification of genetic diversity of the strains at the intraspecies level (Laguerre et al., 1997). The rep-PCR assays showed a high genetic diversity, and the isolates clustered in five main clusters; although the clustering obtained by this fingerprinting approach does not necessarily warrant an inherent phylogenetic value.

The strains were authenticated as rhizobia by two methods. The first method consisted in the determination of *nodC* nodulation gene by PCR. After amplification using nodCFn-nodCi primers, the 930 bp characteristic band was obtained with only 24. The PCR method warrants a fast way to screen the symbiotic bacteria among large number of isolates, from which the nodulation was confirmed by the second method, i.e., the inoculation of the original host. All the isolates that possess nodulation genes were able to form root nodules once re-inoculated on *R. monosperma* seedlings in axenic conditions, while isolates that did not possess *nodC* gene were not.

The isolation of 20 non-nodulating bacteria from nodules reveals the presence of high number of endophytes among the isolates but this is very common in Mediterranean wild legumes due to the presence of several endophytes that can co-inhabit nodules with rhizobia (Muresu et al., 2008).

### Tolerance to Heavy Metals

**FIGURE 2:**
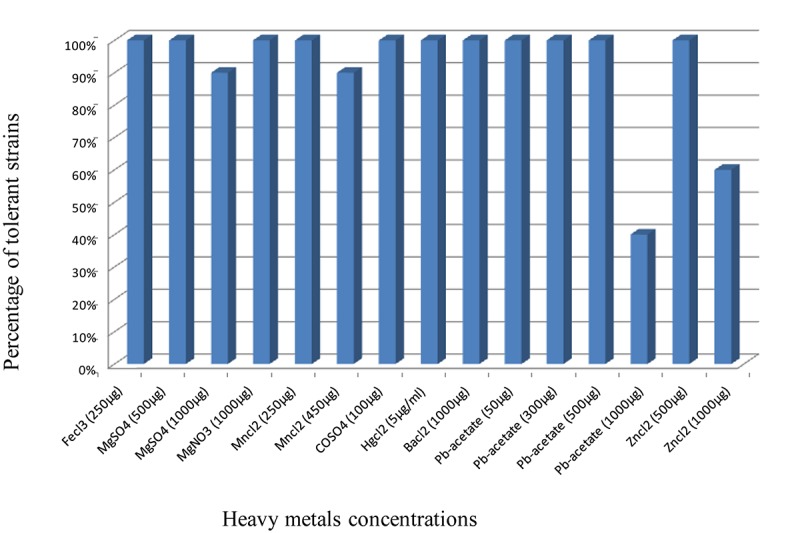
Tolerance of the strains to different heavy metals. Results are presented as percentages of the strains able to grow on the used concentrations of heavy metals.

All the strains were tolerant to Pb-acetate until 500 μg/ml, ZnCl_2_ (500 μg ), BaCl_2_ (1000), HgCl_2_ (5), as well as CoSO_4_ (100), MnCl_2_ (250), MgNO_3_ (1000), FeCl_3_ (250), and MgSO_4_ (500). Out of 24 isolates, 10 grew on TY supplemented with 1000 μg/ml of Pb-acetate, and 15 grew in presence of 1000 μg of Zinc, whereas only one strain was unable to grow on MgSO_4_ (1000 μg) and MnCl_2_ (450 μg) (Figure 2). These results show the high level of strains tolerance to heavy metals.

### Phylogenetic Analysis of 16S rDNA

From the selected strains we retained two strains on the basis of the rep-PCR groupings, for further analysis.

**FIGURE 3:**
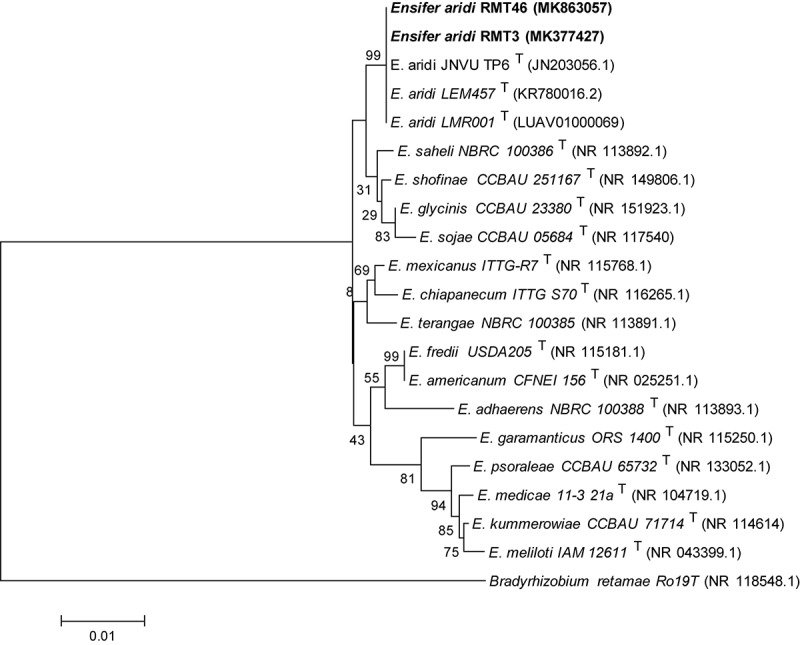
Neighbor-joining phylogeny of 16S rRNA gene sequences of strains from *R. monosperma* nodules and *Ensifer* representative species. The significance of each branch is indicated by a bootstrap value calculated for 1000 subsets.

A phylogenetic tree based on the sequences of the rDNA showed that the two representative strains were 100% related to *Ensifer aridi* strains, recovered from Asian, African, and American deserts (Le Quéré et al., 2017). Therefore, we considered that all our strains belong to the genus *Ensifer* (Figure 3).

### Housekeeping Genes Phylogeny

**FIGURE 4:**
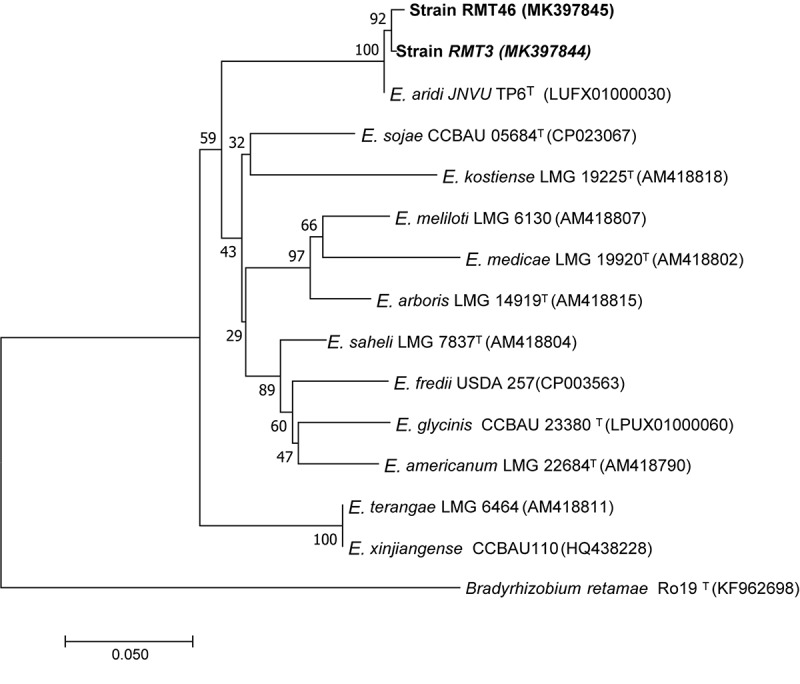
Neighbor-joining phylogeny of *gyrB* gene sequences of *R. monosperma* microsymbionts and *Ensifer* representative species. The significance of each branch is indicated by a bootstrap value calculated for 1000 subsets.

**FIGURE 5:**
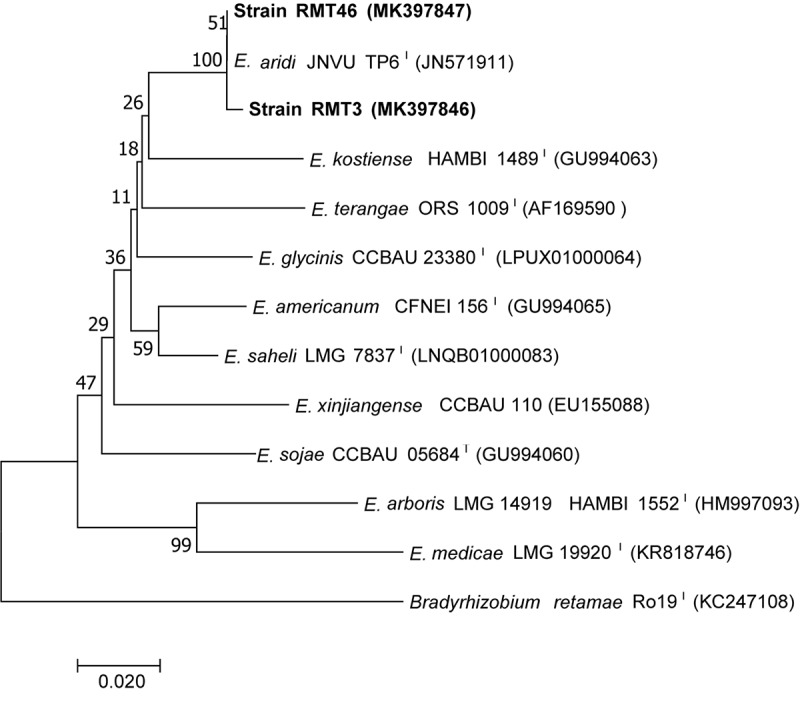
Neighbor-joining phylogeny of *glnII* gene sequences of *R. monosperma* microsymbionts and *Ensifer* representative species. The significance of each branch is indicated by a bootstrap value calculated for 1000 subsets.

**FIGURE 6:**
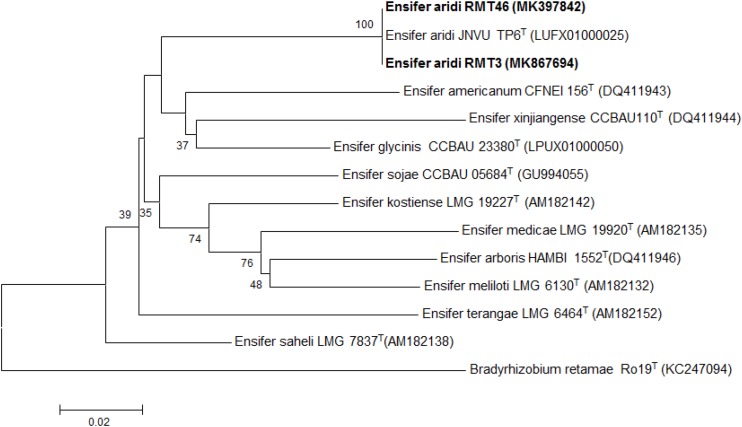
Neighbor-joining phylogeny of *recA* gene sequences of *R. monosperma* microsymbionts and *Ensifer* representative species. The significance of each branch is indicated by a bootstrap value calculated for 1000 subsets.

The results obtained showed that the *gyrB* sequences of the RMT3 and RMT46 strains have a similarity of 99.64 and 99.32%, respectively, with *E. aridi* strain JNVUTP6 (Figure 4). Similar results were obtained with *glnII* sequences which showed a similarity of 99.62 and 100% with *E. aridi* strain JNVU TP6 (Figure 5). The recA sequences of the two strains have 99.47% similarity with *E. aridi* strain JNVU TP6 (Figure 6).

**FIGURE 7:**
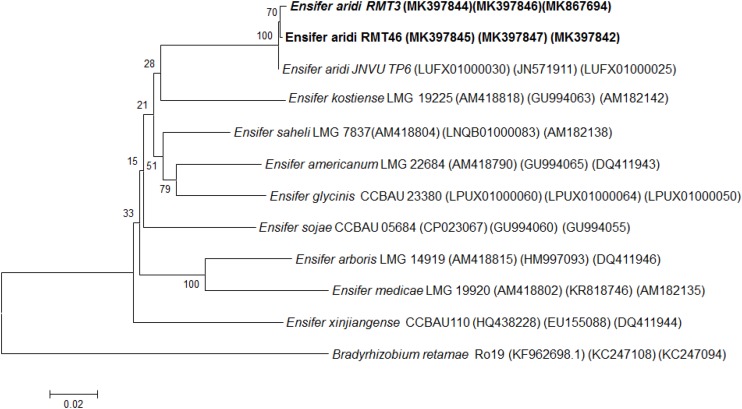
Neighbor-joining phylogeny of members of the genus *Ensifer*, based on a concatenated alignment of *gyrB*, *glnII*, and *recA* gene sequences. The significance of each branch is indicated by a bootstrap value calculated for 1000 subsets.

Analysis of the concatenated sequences of the *recA*, *glnII*, and *gyrB* housekeeping genes (Figure 7) showed that the two strains were related to *E. aridi* strain JNVUTP6 with 98.91–99.61% of similarity.

### Phylogenic Analysis of Symbiotic Genes

Bacterial nodulation (*nod*) genes are involved in the production of Nod factors which act as specific signals triggering nodule formation. The *nodA*, *nodB*, and *nodC* genes are conserved in all nodulating rhizobial strains. They are essential for the synthesis of the lipo-oligosaccharide backbone. The *nodA* gene codes for a 21.8-kDa acyl-transferase and determines the type of the N-acyl substitution transferred into the oligosaccharide backbone of the Nod factor and plays a critical role in making this distinction (Suominen et al., 2001). Thereby, the function of the NodA protein specifies the Nod factor structure and the host range (Ritsema et al., 1996). NodC is an N-acetylglucosaminyl transferase (chitin synthase) that produces chitin oligosaccharides (Machida et al., 2001). Ascertaining the presence of *nodC* and *nodA* genes copy is an important requirement for attributing symbiotic properties to a nodule isolate.

**FIGURE 8:**
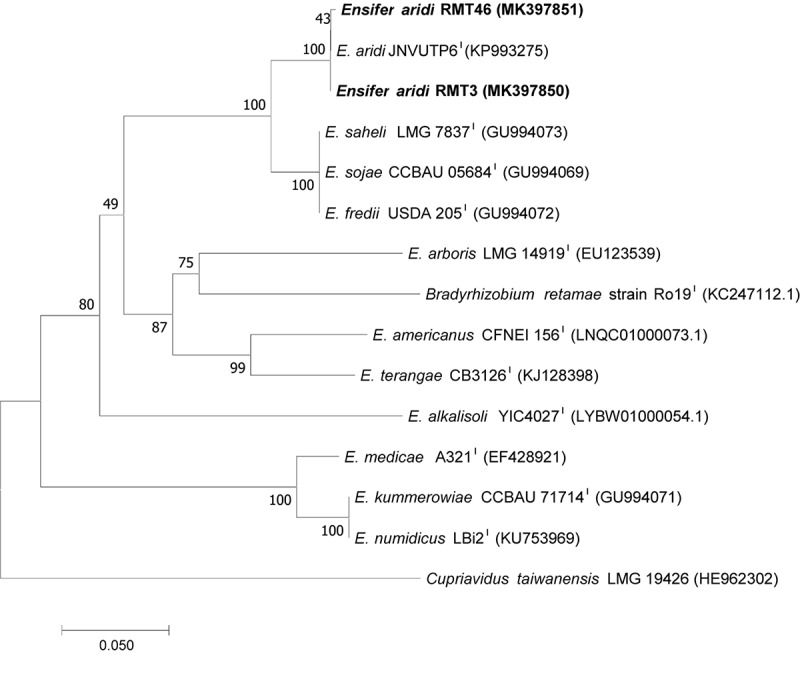
Neighbor-joining phylogeny of *nodC* gene sequences of *R. monosperma* and *Ensifer* representative species. The significance of each branch is indicated by a bootstrap value calculated for 1000 subsets.

To analyze the symbiotic genes diversity in our strains, we amplified and sequenced the two symbiotic genes *nodA*, *nodC*, and *nifH*, the nitrogen fixation gene, using specific primers. Sequencing of the *nodC* gene and phylogenetic analysis permitted the clustering of the two representative strains RMT3 and RMT46 with *E. aridi* strain jnvuTP6 with a similarity of 100 and 99.85%, respectively (Figure 8).

**FIGURE 9:**
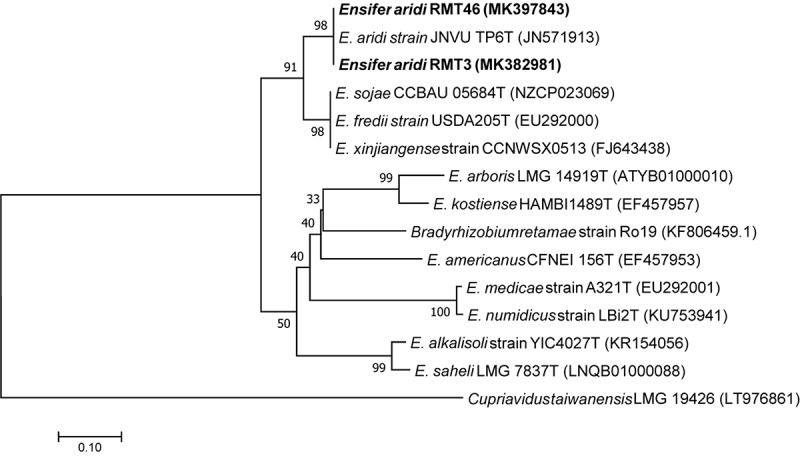
Neighbor-joining phylogeny of *nodA* gene sequences of *R. monosperma* microsymbionts and *Ensifer* representative species. The significance of each branch is indicated by a bootstrap value calculated for 1000 subsets.

Amplification of the *nodA* gene produced a band around 640 bp. The Neighbor joining phylogenic tree generated by *nodA* sequences showed that the two representative strains formed a group with *E. aridi* strain JNVU TP6 with 99.82% similarity with RMT3 and RMT46 (Figure 9).

**FIGURE 10:**
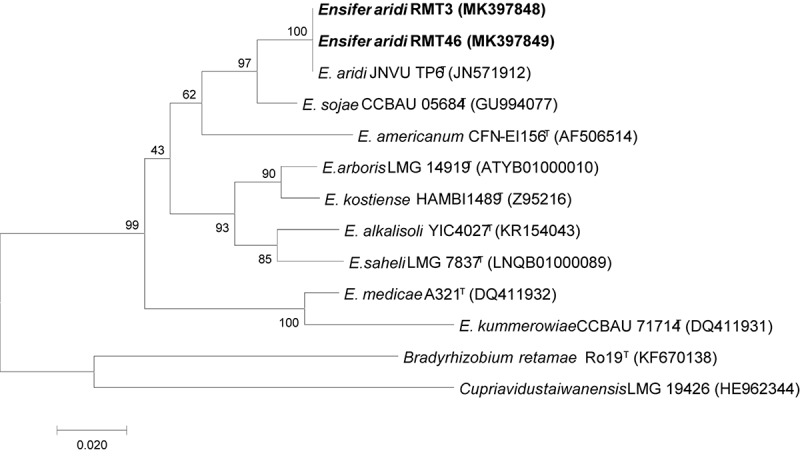
Neighbor-joining phylogeny of *nifH* gene sequences of *R. monosperma* microsymbionts and *Ensifer* representative species. The significance of each branch is indicated by a bootstrap value calculated for 1000 subsets.

The *nifH* gene sequences analysis showed that the two representative strains have a high similarity of 99.38% with *E. aridi* JNVUTP6 (Figure 10).

**FIGURE 11:**
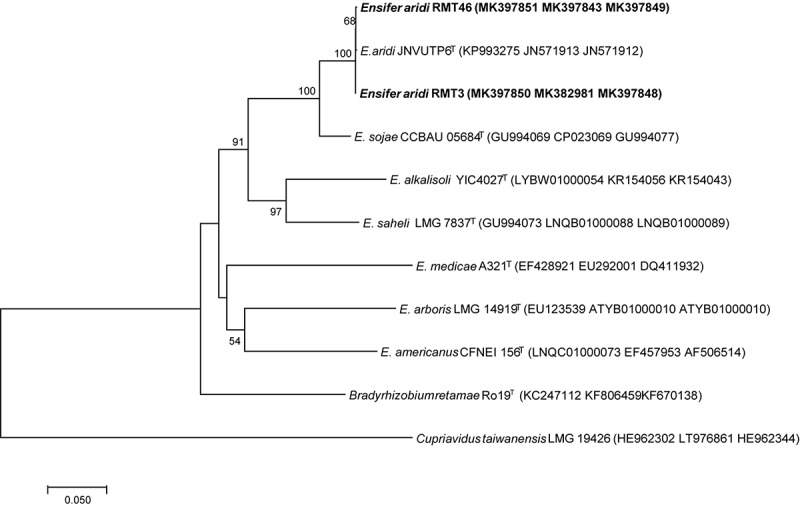
Neighbor-joining phylogeny of *R. monosperma* strains as well as members of the genus *Ensifer*, based on a concatenated alignment of *nodC*, *nodA*, and *nifH* gene sequences. The significance of each branch is indicated by a bootstrap value calculated for 1000 subsets.

The phylogenic analysis of the three symbiotic genes concatenated sequences confirmed the results of 16S rDNA sequencing and housekeeping genes (*gyrB*, *glnII*, and *recA*). These results showed that the two strains are related to *E. aridi* strain JNVUTP6 (Figure 11).

## Discussion

The Eastern area of Morocco and particularly the region of Touissit are known for their hard weather conditions with low and random rainfall as well as a very cold winter and a hot summer. Furthermore, the studied site is located on the lead mine dikes that have been abandoned since 2002 (Smouni et al., 2010). These tons of heavy metal scrap cannot be removed or cleaned up and phyto-stabilization is the only effective way to limit the damages to the environment. Phytostabilization is generally used for soils fixation to prevent the movement of minerals to the nearby villages and population. On this site, several trials of phytostabilization have already been tried without much success, because of the type of introduced plants which are not adapted to the climatic and metallic stresses (Argane et al., 2014).

We preferred the use of nitrogen-fixing leguminous trees for the phytostabilization of these rejects, which are dangerous for humans and the environment. We chose *R. monosperma*, which is known for its ability to establish nitrogen-fixing symbiosis with rhizobia and also for its ability to grow in harsh environments (Hannane et al., 2014). However, extreme conditions may limit the nitrogen fixation process (Berrada et al., 2012), which urges the need to search for the best symbiotic partners that can tolerate these stresses.

The majority of studies done on *Retama* rhizobia have shown that they belong to the genus *Bradyrhizobium* (Hannane et al., 2014). Guerrouj et al. (2013b) described a new species of *Bradyrhizobium* that nodulates *R. monosperma* and *R. sphaerocarpa* in the Eastern region of Morocco and Southern Spain, which they called *B. retamae*. Rodríguez-Echeverría et al. (2014) reported the similarity of some strains nodulating *R. sphaerocarpa* with *B. canariense*, *B. cytisi*, *B. elkanii*, *B. pachyrizi*, and *B. retamae*. More recently, a new species that nodulates *R. sphaerocarpa* in Algerian soils have been described by Ahnia et al. (2018). However, some years ago, Mahdhi et al. (2008) isolated some fast-growing bacteria from root nodules of *R. raetam* in South-western Tunisia belonging to different taxa and whose 16S rDNA was affiliated to *E. kostiense*, *E. meliloti*, *R. giardinii*, *R. leguminosarum*, *R. sullae*, and other *Rhizobium* spp.

In this work we isolated 44 bacteria from the nodules of *R. monosperma* grown in the soils of the lead-zinc abandoned mine of Touissit. We found that only 24 isolates were true rhizobia and were able to renodulate their original host and possess the *nodC* gene. The 20 isolates were considered as endophytes unable to nodulate *R. monosperma* seedlings in axenic conditions. The genetic diversity as assessed by REP-PCR showed that the isolates were regrouped in 10 different fingerprints meaning that we have 10 strains (Figure 1).

All the 10 strains were tolerant to different concentrations of the tested heavy metals. Two strains were selected on the basis of their tolerance to 1000 μg/ml of Pb-acetate as representatives and retained for further studies. Strain RMT46 is able to grow on TY supplemented with 1 mg/ml of lead, whereas strain RMT3 grew only on 500 μg/ml of this heavy metal. However, the concentration of 500 μg/ml of lead is still considered as a highly toxic concentration for bacterial growth in soils (Tiku et al., 2016; Asad et al., 2018).

The molecular analysis, using 16S rDNA and the three housekeeping genes *recA*, *glnII*, and *gyrB* analysis as well as the three symbiotic genes *nifH*, *nodA*, and *nodC*, showed that our strains are related to *E. aridi*. This newly described species has been isolated in African, Asian and American deserts, from root nodules of *V. gummifera* in soils of Merzouga desert in Morocco (Sakrouhi et al., 2016; Le Quéré et al., 2017), root nodules of *Tephrosia purpurea* in the Thar desert in India (Tak et al., 2016), and root nodules of *Phaseolus filiformis* in the Mexican desert (Le Quéré et al., 2017).

We remarked also that *B. retamae* strain Ro19T *nodC* and *nodA* sequences were related to *Ensifer* species that nodulate members of *Acacia* genus in Africa and America (de Lajudie et al., 1994; Nick et al., 1999; Toledo et al., 2003; Figures 8, 9). However, the *nifH* sequences were completely different (Figure 10). This would suggest some lateral transfer of the symbiotic island between *B. retamae* and *E. aridi*.

The presence of excessive levels of heavy metals in soil significantly changes the floristic composition of sites. The deleterious effects of heavy metals on nodulation and nitrogen fixation may be due to their inhibitory effects on the growth and activity of both symbionts. When present in excess, heavy metals may also delay the nodulation process, limit the uptake of water and nutrients by plants and affect concomitantly the health of plants (Terry, 1981; Karpiscak et al., 2001). However, *R. monosperma* supports excessive levels of metals as it grows wildly in heavy metals polluted areas (Belabed et al., 2014).

Moreover, the strains of *E. aridi* isolated from nodules of *R. monosperma* in this work are tolerant to high concentrations of lead, zinc, copper, and mercury. This selective tolerance is probably due to the pressure exerted by heavy metals on microorganisms (Pereira et al., 2006) and plants. Heavy metals can alter and disrupt growth, morphology, and activities of non-tolerant rhizobia (Ahmad et al., 2012). This ability allows them to grow and fix nitrogen in symbiosis with their host plant in the mining environment of Touissit. The plant thus becomes able to establish, fix the soil, and thus participate to the phytostabilization of this mining site.

## Conclusion

In this study, we isolated 44 bacteria, from the root nodules of *R. monosperma* grown in the Touissit lead mine tailings, in the arid Eastern Morocco. We selected 2 strains representing the 10 efficient rhizobial strains for molecular analysis and found they are related to *E. aridi*, a species that is adapted to the extreme deserts conditions and improves the resilience of legumes in these hostile environments.

We consider that *R. monosperma* and *E. aridi* would be very good candidates for the restoration of this mining site. It will thus be possible to propose an inoculum based on these strains to the National Forest authorities (*Haut-Commissariat aux Eaux et Forêts et Lutte Contre la Désertification*) to be used for the inoculation of *R. monosperma* plants during reforestation and phytostabilization programs and campaigns.

## Data Availability

The raw data supporting the conclusions of this manuscript will be made available by the authors, without undue reservation, to any qualified researcher.

## Author Contributions

MM-EI, EB, and HA contributed to the conception and design of the work. HL, SA, OB, and SE contributed to the realization of the experimentations and first treatments of the results, at different levels. MM-EI and HL conducted the analysis and interpreted the data. HL and MM-EI wrote the first draft of the manuscript. All authors contributed to revision of the manuscript.

## Conflict of Interest Statement

The authors declare that the research was conducted in the absence of any commercial or financial relationships that could be construed as a potential conflict of interest.
